# Sweeping the Floor or Putting a Man on the Moon: How to Define and Measure Meaningful Work

**DOI:** 10.3389/fpsyg.2017.01658

**Published:** 2017-09-29

**Authors:** Jitske M. C. Both-Nwabuwe, Maria T. M. Dijkstra, Bianca Beersma

**Affiliations:** ^1^Organization Sciences, VU University Amsterdam, Amsterdam, Netherlands; ^2^Cordaan, Amsterdam, Netherlands

**Keywords:** definition, meaningful work, meaningfulness, scales

## Abstract

Meaningful work is integral to well-being and a flourishing life. The construct of “meaningful work” is, however, consistently affected by conceptual ambiguity. Although there is substantial support for arguments to maintain the status of conceptual ambiguity, we make a case for the benefits of having consensus on a definition and scale of meaningful work in the context of paid work. The objective of this article, therefore, was twofold. Firstly, we wanted to develop a more integrative definition of meaningful work. Secondly, we wanted to establish a corresponding operationalization. We reviewed the literature on the existing definitions of meaningful work and the scales designed to measure it. We found 14 definitions of meaningful work. Based on these definitions, we identified four categories of definitions, which led us to propose an integrative and comprehensive definition of meaningful work. We identified two validated scales that were partly aligned with the proposed definition. Based on our review, we conclude that scholars in this field should coalesce rather than diverge their efforts to conceptualize and measure meaningful work.

## Introduction

There is a famous story about President John F. Kennedy’s first visit to NASA’s headquarters back in 1961. During his visit to the NASA space center, as the story goes, President John F. Kennedy noticed a janitor carrying a broom. He interrupted his tour, walked over to the man and asked: “What are you doing?” “Well, Mr. President,” the janitor responded, “I’m helping to put a man on the moon.”

This story brings to life an image of how seeing a bigger purpose for one’s work than just the tasks at hand can make employees more engaged and satisfied in their work. Furthermore, it is easy to imagine that seeing such bigger purpose would serve both their personal and the organizational goals. Indeed, research has linked experiencing work as “meaningful” to individual outcomes, such as work engagement, job satisfaction and motivation, and to organizational outcomes, such as performance (Martela, 2010; Lips-Wiersma and Wright, 2012; Steger et al., 2012). Furthermore, meaningful work is believed to be integral to well-being and a flourishing life (Rosso et al., 2010; Veltman, 2016). Flourishing refers to the experience of a sense of happiness (Veltman, 2016). Without meaningful work, it is unlikely that a person will flourish (Veltman, 2016). Considering these ideas and research findings, it is no surprise that meaningful work is receiving increasing attention from researchers as well as practitioners (Lips-Wiersma and Morris, 2009).

Despite the current interest in meaningful work, there is a lack of consensus as to the nature of the construct. This is undesirable because consensus in a research field is often seen as an essential factor in making scientific progress, and without some minimal level of consensus about paradigmatic approaches and methods, knowledge development in the scientific field cannot occur (Pfeffer, 1993). Adding empirical findings to this perspective, two studies by Lewandowsky et al. (2013) demonstrated that perceived scientific consensus in a field of research is essential for its acceptance by the general public. In their first study on pedestrians in downtown Perth, for example, they found that people were more likely to accept scientific facts when they believed there was consensus within the field of study these facts derived from. In their second study, they found that scientific consensus even improves the acceptance of scientific facts in situations in which social norms are ambiguous, which could cause people to discredit the scientific facts.

In line with these findings, Rolfe-Redding et al. (2012) found, in their detailed analysis of Republican opinion on climate change that perceived scientific consensus is the strongest predictor of acceptance of climate science by policymakers. In their experiment-control study on vaccine safety, Van der Linden et al. (2015) found that highlighting medical consensus on vaccine safety increases perceived scientific agreement, which promotes favorable public attitudes toward vaccination and reduces perceived risk and belief in the (long discredited) autism-vaccine link. In their quantitative study comparing the structure of knowledge in four scientific fields, Lodahl and Gordon (1973) found that high-paradigm fields, which are high in consensus on certain theories and findings, attract more funding. According to Lodahl and Gordon (1973), the reason for this might be that the consensus on certain theories and findings that characterizes high-paradigm fields clarifies directions for further lines of inquiry. Both policymakers and the public, therefore, can be more certain of obtaining results, which leads to more funding being allocated to high-paradigm fields (Lodahl and Gordon, 1973). Highlighting scientific consensus to the general public and policymakers, therefore, is important for public acceptance as well as for receiving financial support.

Other benefits of consensus on paradigmatic approaches and methods in a given scientific field are manifold. Pfeffer (1993), for example, discusses how such approaches and methods are related to lower journal rejection rates and more collaboration on research. Hargens (1988) analyzed journal rejection rates for 30 scientific journals from 1967 to 1983, and found that journal acceptance rates in high-paradigm fields were higher. Pfeffer and Langton (1993) explored the antecedents and circumstances of research collaboration among more than 60,000 faculty members in 303 colleges and universities. They concluded that research collaboration in high-paradigm fields were much more intense than in low-paradigm fields, meaning that there was greater awareness of colleagues’ research projects and there were more joint research projects. They suggested that, as paradigms develop, greater efficiency is achieved through the achievement of consensus and its communication (Pfeffer and Langton, 1993). In other words: if researchers agree on their topics of study, it becomes easier to communicate and collaborate, which, in its turn, leads to scientific progress.

Of course, there are also arguments in favor of not having consensus in a field of study. Lack of consensus, for example, has been found to relate to creativity, inclusiveness, and theoretical and methodological diversity (Pfeffer, 1993). Although we agree that diversity in theoretical and methodological perspectives can be useful for a research field at a given point in time, we would argue that, in light of the arguments and research findings presented earlier, it is very helpful for a scientific field to eventually move toward agreement on definitions of central constructs on the basis of accumulated evidence. Such agreement, on the multidimensional characteristic of meaningful work, for example, can both facilitate efficient communication and effective collaboration among researchers as well as clear communication with practitioners interested in meaningful work.

Furthermore, a sufficient level of agreement over rules for operationalization (for example, multidimensional meaningful work scales) is very useful, as it facilitates comparing results and integrating theoretical perspectives (Pfeffer, 1993). The research field of meaningful work is at the beginning of paradigm development. Reaching consensus on a definition of meaningful work, explaining what it is and what it is not, could enhance knowledge development and communication efficiency in this field, in other fields, and in the public at large. Highlighting scientific consensus on a definition of meaningful work could enhance public acceptance of research findings as well as secure financial support for further research. To advance the field of meaningful work, therefore, instead of developing new theories and definitions, it is important to integrate existing theories and corresponding definitions of meaningful work to establish an unambiguous integrative definition of meaningful work. Such a definition can then be employed in different theories and empirical studies in this field.

Another benefit of consensus on a definition of meaningful work is that it opens the way to some agreement over rules for operationalization. Of course, the ambiguity surrounding the definition of meaningful work inhibits comparisons between theories on the construct, but it also has implications for its measurement: if the definition of meaningful work is ambiguous, so will be its measurement. As a result, there are many different instruments to measure meaningful work, which has led to scattered research findings with questionable comparability. As many scales already exist, we will examine currently existing meaningful work scales – rather than develop a new one – to identify universal methodological approaches. Agreement on methodology, as it leads to comparable research findings, will help to advance knowledge in the field.

Reflecting on the origin of the conceptual ambiguity by which the concept of meaningful work is currently affected, several reasons must be considered. One reason is that the meaningful work construct has been examined in many different research contexts. In the empowerment literature, for example, meaningful work is understood as a sub-construct of empowerment (Lee, 2015a) whereas it is understood as an experience or sense of purpose in research on transformational leadership (Martela, 2010; Lee, 2015a).

Another reason for the conceptual ambiguity surrounding meaningful work is that different theoretical frameworks have been proposed as to what it comprises. Based on their review, Lepisto and Pratt (2016) recently proposed to consider meaningful work from either of two perspectives: a realization perspective or a justification perspective. Viewed from the realization perspective, meaningful work is created through fulfillment of needs, motivations, and desires associated with self-actualization. Viewed from the justification perspective, it is created through the subjective experience of the value or worth of one’s work, that is, its higher purpose.

While the two perspectives proposed by Lepisto and Pratt (2016) help to organize the literature on meaningful work, their proposed alternative perspectives appear to be disregarding the multidimensionality of the construct of meaningful work. Recently, there has been more agreement that meaningful work needs to be conceptualized as a multidimensional construct, or as the result of a complex interplay of multiple dimensions (Lips-Wiersma, 2002a,b; Chalofsky, 2003; Lips-Wiersma and Morris, 2009; Rosso et al., 2010; Steger et al., 2012). Despite such agreement, there is no consensus about the exact nature of the different dimensions underlying the concept of meaningful work (Bendassolli et al., 2015). As a result, it has been defined in many different ways, and, as authors have previously noted, there is no generally agreed upon definition (Martela, 2010; Rosso et al., 2010).

According to the definition of Rosso et al. (2010), “meaningful work is work experienced as particularly significant and holding more positive meaning for individuals” (p. 95). As this definition repeats the term to be explained in the phrase that is meant to explain it, it is tautological, and as the concepts of “work” and “positive meaning” are not really explained, the definition does not provide us with a full understanding of the concept of meaningful work.

Addressing the conceptual ambiguity of the construct of meaningful work, Lee (2015a) proposed an alternative definition based on concept analysis. Based on identifying critical attributes of meaningful work in the literature, Lee (2015a) defines meaningful work as follows: “Meaningful work is the discovery of existential meaning from work experience, work itself and work purpose/goals” (p. 2263). In this definition, Lee (2015a) provides an underlying conceptual framework that is believed to bring forth meaningful work (work experience, work itself, and work purpose/goals). Although this definition clarifies what underlying conceptual framework has been used, the framework itself only comprises the work context and omits three other sources of meaningful work (i.e., the self, others, and spiritual life) that were argued by Rosso et al. (2010) to also affect the meaningfulness workers experience in their work. Emphasizing a single source of meaningful work provides an overly simplistic view of how people construct meaningfulness in their work (Rosso et al., 2010). To understand comprehensively how work becomes meaningful, we must take into account the integrative nature of sources of meaningful work (Rosso et al., 2010).

Lee’s (2015a) definition, based on only the work context, therefore, does not establish consensus but rather adds to the discussion on what defines meaningful work. In spite of these recent attempts by Rosso et al. (2010) and Lee (2015a) to improve our understanding of the concept of meaningful work, consensus on how the construct should be defined thus has still not been reached, as currently available definitions are either incomplete or tautological.

In conclusion, we argue that conceptual clarity about the construct of meaningful work is essential to the field and its progress (Pfeffer, 1993). The main focus in the present study therefore is on the definition and operationalization of the construct of meaningful work. We will review previous definitions of meaningful work in order to arrive at a more integrative definition. We will also review scales designed to measure the construct to evaluate (a) the extent to which they fit this integrative definition and (b) their psychometric qualities.

To define meaningful work, we first have to delineate what “work” is. Our perspective to this issue is, firstly, an ethical one: we consider only activities that are morally worthy and legal. We do not consider immoral or illegal activities, drug dealing for example, to fall under the definition of work, because they are not lawful or morally worthy. Secondly, we focus on paid work. Although Veltman (2016) argue to define work as a holistic concept encompassing also tasks and activities in the context of caring for family members, or volunteering work, there are important differences between paid work that is performed on an occupational basis and other types of tasks. Arguably, in the contexts of family life and volunteering, the level of personal and autonomous choice involved for engaging in tasks is higher. This does not mean that paid workers are not doing what they like for a living, but most likely, a reason for them to work, at least in part, is because they need to earn money. Therefore, there arguably are differences in personal and autonomous choice between paid and unpaid work, and these differences are likely to influence the meaningfulness people experience when engaged in their tasks. Research on meaningful work or non-work tasks should therefore be viewed in the specific context in which it is performed. In the current study we focus on the context of paid work. In conclusion, we define tasks and activities as work when they concern paid activities on an occupational basis that are lawful as regards their nature (see also Lepisto and Pratt, 2016).

Having specified how we define as “work,” we focus on the meaningfulness of work. Our purpose is twofold. First, we aim to develop an integrative definition of meaningful work. Second, we aim to produce a uniform and unequivocal operationalization of the construct of meaningful work to be used in empirical studies. In order to do so, we built on the work of Rosso et al. (2010) who provided an extensive literature review of empirical and theoretical articles on meaningful work. Their analysis revealed that, although meaningful work was examined in different contexts, two key issues are central in understanding what makes work meaningful: first, *what* factors influence the experience of meaningful work, that is, its sources, and second, *how* work becomes meaningful, that is, its underlying psychological and social mechanisms. These two issues were often entangled in the past (Lips-Wiersma and Morris, 2009; Steger et al., 2012).

Below, we will introduce our first literature review, focusing on definitions of meaningful work. After discussing the results of this review and integrating the results into an integrative definition of meaningful work, we will introduce our second literature review, focusing on scales designed to measure meaningful work. Finally, we will discuss the outcomes of our review of measurement scales and formulate several directions for future research.

## Study 1: Defining Meaningful Work

The purpose of the first literature review was to analyze the concept of meaningful work in the literature, to provide an overview of the existing definitions, and to propose an integrative and comprehensive definition based on this overview. Researchers have often used “meaningfulness” and “meaning” interchangeably. Following Rosso et al. (2010), we have differentiated between these concepts in this article. Meaningfulness refers to the perceived level of significance of one’s work (Monnot and Beehr, 2014). As such, a single work event may be experienced as extremely significant, or meaningful, by one worker and as not significant at all, or not meaningful, by another worker (Rosso et al., 2010).

Meaning, on the other hand, is the outcome of having made sense of work. As Rosso et al. (2010, p. 94) put it: “meaning is an individual interpreting what her work means, or the role her work plays, in the context of her life (e.g., work is a pay check, a higher calling, something to do, an oppression).” We have focused on the literature that addresses meaningful work as a multidimensional construct. The meaning of work is outside the scope of this review.

### Methods

#### Search Strategy

In order to provide an overview of the existing definitions of meaningful work, we conducted a literature review in June 2016 via Internet using electronic databases and a search engine. We used the following electronic databases: Medline (Ovid), Sciencedirect, PsycINFO, and SSRN: Social Science Research Network, in order to obtain articles related to meaningful work studies from distinct areas, including humanist studies, psychology, and organizational science. As search terms in the electronic databases, we used the combined keywords “meaningful work” and “definition” and the combined keywords “meaningful work” and “concept.” We used the Google Scholar search engine at http://www.scholar.google.com for our Internet search. As search terms for the search engine, we used “allintitle: meaningful work.” When searching for articles, we did not put limits on year of publication.

#### Inclusion and Exclusion Criteria

We applied two inclusion criteria. First, in line with the emerging conceptualization of meaningful work as a multidimensional construct (see, e.g., Lips-Wiersma, 2002a,b; Chalofsky, 2003; Rosso et al., 2010; Steger et al., 2012), we selected articles that conceptualized meaningful work as a multidimensional construct. Second, we selected only articles that were written in English. In order to better understand the concept of meaningful work and following Lee (2015a), we applied the following exclusion criterion: articles that did not provide any reasoning for the definition they used or did not provide an underlying conceptual framework of meaningful work.

#### Data Extraction and Analysis

The initial screening of articles was done by reading the titles and abstracts. Titles were reviewed, and abstracts were retrieved if potentially relevant information was identified in the title. Then, abstracts were reviewed, and full texts were retrieved if potentially relevant information was identified in the abstract. Furthermore, reference lists of retrieved full texts were scanned, and Web of Science (a citation index) was searched for related literature that had previously been missed or omitted.

A four-step inductive content analysis procedure was used. The objective was to let the themes emerge from the data rather than predetermine them. This inductive process was considered appropriate because of the conceptual ambiguity surrounding the concept of meaningful work. If knowledge is fragmented, the inductive approach is recommended (Elo and Kyngäs, 2008). The first step included the recording of the identified definitions and framework of meaningful work. Next, themes within the recorded definitions were identified and coded. The third step involved comparing themes and naming emerging categories. A particular category was formed when a theme occurred more than once across the definitions. The following step included exploring the properties of categories, identifying relationships between categories and uncovering patterns to draw conclusion for an integrative definition. The goal was to draw an integrative definition from all the identified categories. The first author performed the steps first. Thereafter, all the steps were discussed among the three authors.

### Results

The search yielded 1,990 articles. Screening of titles and abstracts resulted in 1,919 articles being excluded. One article was identified through the reference list of a retrieved full text. The full texts of 72 articles were read, and 14 definitions of meaningful work were identified (Bowie, 1998; Chalofsky, 2003; Pratt and Ashforth, 2003; Miller, 2008; Lips-Wiersma and Morris, 2009; Martela, 2010; Rosso et al., 2010; Fairlie, 2011; Lips-Wiersma and Wright, 2012; Pavlish and Hunt, 2012; Steger et al., 2012; Robertson, 2013; Bendassolli et al., 2015; Lee, 2015a; Allan et al., 2016). Some authors used the same multidimensional framework for meaningful work – for example, the Kantian model, in which meaningful work can be created by certain objective normative ethical work characteristics (Bowie, 1998; Pavlish and Hunt, 2012) – but provided different definitions.

We identified 14 unique definitions. The content analysis approach led us to identify four categories of definitions: (1) “positive significance or purpose,” the largest category with seven definitions of meaningful work, focusing on the experience of positive significance or purpose through work; (2) “constituents of meaningful work,” with four definitions, focusing on what meaningful work consists of; (3) “fit,” with two definitions, focusing on the fit between the individual and his/her work; and (4) “fulfillment,” with two definitions focusing on fulfilling a certain need or dimension. Looking closer at the categories and relationships between categories, we noticed that most definitions fell within the category of “positive significance or purpose.” Two out of seven definitions in this category refer to Rosso colleague’s definition while adding some extra explanation. However, these two definitions are still tautological: they essentially define meaningful work as work that generates meaning. Looking for the patterns and relationship between the categories we found that the categories ‘constituents of meaningful work’ and ‘fulfillment’ define meaningful work in terms of ‘how’ work becomes meaningful. The category ‘positive significance or purpose’ defines meaningful work in terms of experiences. The ‘fit’ category defines meaningful in terms of ‘when’ work becomes meaningful. The definition ‘meaningful work is an individual subjective experience of the existential significance or purpose of work’ was most aligned with the other definitions in this category and therefore used as part of the integrative definition. Within the other categories, we could not find a definition that aligned with all the others in that category. It was therefore decided to use the categorical names in the integrative definition.

The findings are presented in **Table 1**.

**Table 1:** Overview of definitions and framework of meaningful work (MW).

Reference	Framework meaningful work	Definition
**Category 1: positive significance and/or purpose**		
Rosso et al., 2010	Based on the literature, Rosso et al. (2010) offer four main pathways to the creation or maintenance of meaningful work: individuation, contribution, self-connection, and unification.	MW is work experienced as particularly *significant* and holding more *positive* meaning for individuals.
Robertson, 2013	Follows the framework of Rosso et al. (2010).	This paper adopts Rosso et al.’s (2010) definition of MW: “work experienced as particularly *significant* and holding more *positive* meaning for individuals,” and adds: where “meaning” is the result of making sense of something within the context of one’s life.
Steger et al., 2012	Based on research on calling and MW, Steger et al. (2012) propose a three-dimensional model of MW: (1) positive meaning in work; (2) meaning making; and (3) greater good. Positive meaning is the subjective experience that what one is doing has personal significance. Meaning making is the experience that work attributes to meaning in life as a whole. Greater good is the desire to make a positive impact on others.	This paper adopts Rosso et al.’s (2010) definition of MW: work that is both *significant* and *positive* in valence (meaningfulness) and adds: the positive valence of MW has a eudemonic (growth- and purpose-oriented) rather than hedonic (pleasure-oriented) focus.
Martela, 2010	Based on Baumeister’s model for meaning, MW is proposed to consist of four individual elements: the need for purpose, values, efficacy, and self-worth. Work is meaningful when it is able to fulfill one or many of these needs, but the needs individual workers attempt to fulfill through their work depend on their larger life context.	MW is work that offers the worker *positive significance* in life thus contributing to the fulfillment of the human need for meaningfulness.
Lips-Wiersma and Wright, 2012 Lips-Wiersma and Morris, 2009	Based on qualitative psycho-biographical research and action research, Lips-Wiersma (2002a,b) proposes a four-dimensional model of MW: (1) developing and becoming self; (2) expressing full potential; (3) unity with others; (4) service to others. Moreover, Lips-Wiersma (2002a,b) argues these dimensions are on three existential continuums: (1) individual- others; (2) doing and being; and (3) reality and inspiration. The individual-other continuum refers to fulfilling the needs of oneself and others. The doing-being continuum refers to fulfilling the need to do and the need to be. The ‘need to be’ can be described in terms of self-actualization or one’s own identity. The reality-inspiration continuum refers to coming to terms with an imperfect self in an imperfect world (reality-check) at one end, and the inspiration or hope to improve oneself and the conditions for others at the other end. The dimensions need to be balanced on these continuums in order to experience MW. MW is thus a seven-factor construct, consisting of four dimensions on three continuums.	MW is an individual subjective experience of the existential *significance* or *purpose* of work.
Pratt and Ashforth, 2003	Based on identity theory and social identity theory, Pratt and Ashforth (2003) propose a four-dimensional model of MW: (1) one’s role and (2) one’s membership leads to (3) one’s identity, which leads to meaningfulness through sense-making.	Work and/or its context are perceived by its practitioners to be, at minimum, *purposeful and significant.*
Chalofsky, 2003	Based on the literature, Chalofsky proposes that meaningful work consists of three dimensions: work itself, a sense of self, and a sense of balance.	MW *gives essence* to what we do and brings a *sense of fulfillment* to our lives.
**Category 2: constituents of meaningful work**		
Allan et al., 2016	MW is experienced in engaging in intrinsically motivated work behavior, thereby creating congruence between work behaviors and one’s self-concept, which results in feelings of meaningfulness. MW is considered as a key outcome of self-determination. Based on Self-Determination Theory and the Psychology of Working Framework, intrinsically motivated work is achieved through autonomy, relatedness, and competence, survival/power needs, relational needs, and self-determination needs.	MW is the subjective experience that one’s work *has significance, facilitates personal growth, and contributes to the greater good.*
Lee, 2015a	Based on concept analysis, Lee proposes a four-dimensional model for MW: (1) experienced positive emotion at work; (2) meaning from work itself; (3) meaningful purpose and goals of work; and (4) work as a part of life toward meaningful existence. MW is defined as the discovery of existential meaning from work experience, work itself, and work purpose/goals. Experienced positive emotion at work reflects subjective positive experience including meaningfulness, a sense of worth, and self-fulfillment, when employees have meaning in work. Meaning from work itself indicates work attributes that provide meaning, such as work significance, work values, and work orientation. Meaningful purpose and goals of work indicate that meaning in work can be derived from knowing what employees want to be and do in the workplace. Work as a part of life toward meaningful existence reflects the impact of meaning in work on one’s personal life, a personal reason for existence, and an authentic self. The instrument integrates the perspectives of the experience of meaningful work and work as an attribute of existential meaning.	MW is the discovery of existential meaning from experiencing *positive emotion, finding meaning from work, and pursuing purpose or goals in the workplace.*
Bowie, 1998	Based on Kant’s Moral Theory, Bowie describes six characteristics of meaningful work: (1) Meaningful work is work that is freely entered into. (2) Meaningful work allows the worker to exercise her autonomy and independence. (3) Meaningful work enables the worker to develop her rational capacities. (4) Meaningful work provides a wage sufficient for physical welfare. (5) Meaningful work supports the moral development of employees. (6) Meaningful work is not paternalistic in the sense of interfering with the worker’s conception of how she wishes to obtain happiness.	MW is work that is *freely entered into, that allows the worker to exercise her autonomy and independence, that enables the worker to develop her rational capacities, that provides a wage sufficient for physical welfare, that supports the moral development of employees, and that is not paternalistic in the sense of interfering with the worker’s conception of how she wishes to obtain happiness.*
**Category 3: fit perspective**		
Bendassolli et al., 2015	Based on the work of Morin and Dassa (2006), MW is viewed as a three-dimensional model: (1) the significance of work; (2) individual’s orientation; and (3) coherence or harmony in work. The significance of work refers to the significance an individual attaches to work and the representations and values the individual attributes to it. The individual’s orientation refers to the individual’s inclination regarding work, the individual’s objective at work, and the plans that guide the individual’s actions. Coherence refers to the coherence or harmony between the individual and his or her work. The MWS measures the effect of this coherence based on the identification of six general characteristics of MW: work utility; moral correctness; autonomy; development and learning; quality of working relationships; and expressiveness and identification at work. Work Utility assesses the social function of work and its impact on people and society. Moral Correctness assesses the perception of justice and fairness in labor relations. Autonomy evaluates the perception of the subject’s freedom to organize work his or her own way. Development and Learning addresses how much work enables growth and skill development. Quality of Work Relationships evaluates the work environment, interactions, and companionship at work. Expressiveness and Identification at work assesses how much work enables expressiveness and identification at work.	MW is *an effect of the coherence* between the characteristics one pursues and the characteristics he/she identifies at the work he/she does.
Pavlish and Hunt, 2012	Following Bowie’s characteristics of meaningful work based on Kantian theory (Bowie, 1998).	Meaningful work is *the value of work goals judged in relation to an individual’s own ideals and passions* and specifically as work that “gives essence to what we do and brings fulfillment to our lives.”
**Category 4: fulfillment**		
Miller, 2008	Based on the model of Chalofsky (2003).	MW is the *ability to earn a living doing that which satisfies an individual’s psychological, spiritual, and social sense of purpose and contribution*.
Fairlie, 2011	Based on Baumeister’s model of meaning, four needs are identified for meaningful work: purpose (including goals and fulfillments), values, efficacy, and self-worth.	Meaningful work is defined as *aspects of one’s job or work environment that facilitate the attainment or maintenance of one or more dimensions* of meaning.


### Discussion

At the beginning of this article, we observed that there is no universally accepted definition of meaningful work. For this field to develop and grow, therefore, it is important to reach some level of consensus, which the review of Rosso et al. (2010) has not established. Indeed, our Study 1 demonstrated that 14 different definitions could be identified in the literature. Within these 14 definitions, we identified four categories. Most definitions fell within the category of “positive significance or purpose,” perhaps because of the influence of the review by Rosso et al. (2010).

Unexpectedly, Study 1 demonstrated that research on meaningful work rarely defines “work.” This omission has also been noted by Lepisto and Pratt (2016) in their review of the nature of meaningful work. If a definition of work is lacking (Chalofsky, 2003; Lepisto and Pratt, 2016), the term may refer to different entities, such as work activities and tasks, a collection of tasks that make up a job, or one’s career as one of life’s dimensions (Lepisto and Pratt, 2016).

In order to bring consensus to the field of meaningful work, to conceptualize meaningful work more comprehensively, and to avoid tautology, we propose to define it on the basis of all the four categories we derived from our review, as follows: meaningful work is the subjective experience of existential significance resulting from the fit between the individual and work. “The subjective experience of existential significance” refers to the process of personally perceiving work as contributing to, or making sense of, one’s reason of existence in the world. “Resulting from the fit” refers to the fulfillment of certain dimensions – inherent in every human being – through or in work. These dimensions should be further defined by an underlying conceptual framework.

## Study 2: Meaningful Work Scale

The purpose of the second literature study was to evaluate meaningful work scales in light of the definition proposed above and to assess their psychometric characteristics, by providing an overview and examining validated scales.

### Methods

#### Search Strategy

In order to provide an overview of meaningful work scales, we conducted a literature review via Internet in June 2016 using the same electronic databases and search engine as for Study 1. We used the search terms “meaningful work scale” and “measuring meaningful work.” The search was done without putting limits on year of publication.

#### Inclusion and Exclusion Criteria

We applied two inclusion criteria: (1) empirical studies that used, validated, or adapted instruments that measured meaningful work as a multidimensional construct; and (2) studies written in English. We applied three exclusion criteria: studies in which (1) no definition of meaningful work was provided; (2) the method of measuring meaningful work was not described; or (3) meaningful work was measured one-dimensionally. We chose these exclusion criteria to focus exclusively on validated instruments that measured meaningful work as a multidimensional construct.

#### Data Extraction and Quality Assessment

For the data extraction process, we followed the same steps as described in Study 1. In one instance, the authors were contacted for a copy of the text when the full text could not be retrieved.

Following extraction, the characteristics of studies were recorded, including definition of meaningful work, operationalized dimensions of meaningful work, study characteristics, scale characteristics, and psychometric characteristics. To evaluate measurement alignment between the scales and our proposed definition, we checked the scales for face validity (Drost, 2011). We specifically checked whether the scales: (1) captured the experience of meaningful work and (2) captured the features of both work and the individual contributing to fit. Methodological quality assessment of the scales was done by describing the sample characteristics, reliability, and measurement validity of each scale. Sample characteristics were described by examining study size. Reliability assessment addressed whether the internal consistency of the subscales was sufficient. Following Peterson (1994), internal consistency was considered sufficient if Cronbach’s Alpha was 0.70 or higher.

We were interested in subscale reliability because the scales were developed on the basis of *a priori* multidimensional frameworks of meaningful work. Hence, each dimension should be measured by its own reliable subscale.

Following Campbell and Fiske (1959), we addressed the construct validity of the included scales by describing their convergent and discriminant validity. We addressed the convergent validity of the scales by describing the correlations between the scale and other scales measuring similar constructs (for example, “calling,” conceptualized as a specific purpose to serve some greater good; Lips-Wiersma and Wright, 2012; Steger et al., 2012). We addressed the discriminant validity of the scales by describing the correlations between the scale and other scales measuring different constructs (for example, meaning in life; Lips-Wiersma and Wright, 2012). Following the recommendations of Hu and Bentler (1999), we also assessed the results of the confirmatory factor analyses by describing the comparative fit index (CFI) and root mean square error of approximation (RMSEA) of the instruments.

### Results

The search yielded 212 articles. Screening of titles and abstracts resulted in 101 articles being excluded because they only addressed meaningful work without measuring it.

The full texts of 111 articles were read, and seven instruments were initially identified that measured meaningful work as a multidimensional construct. After closer examination, three instruments were excluded because the scale validation study for one instrument was written in German (Höge and Schnell, 2012); the scale validation study for a second instrument did not include confirmatory factor analysis (Fairlie and Flett, 2004); and the scale for a third instrument was intended to measure the role work plays in the context of life (e.g., work meaning, pay check, or life fulfillment) and significance of work. For this latter part, the authors used a scale that was included in this review, the so-called WAMI scale (Arnoux-Nicolas et al., 2016).

We found that many studies used instruments that measured concepts that were related to the construct of meaningful work or did not address the various dimensions of meaningful work. For example, we found that some instruments measured similar but different concepts, such as the Engagement in Meaningful Work Scale (EMWS; Treadgold, 1999). The EMWS is designed to measure the degree to which people perceive their work as something they are intrinsically motivated to do and also feel called upon to do by their inner guidance. It measures the concepts of calling and intrinsically motivating work. Although these concepts are very similar to meaningful work, they are conceptually different. Callings are often seen as being related to one’s authenticity (Rosso et al., 2010). Research on calling suggests that when work provides individuals with opportunities to pursue their identified specific purpose, work is considered to be more meaningful because it is experienced as being personally fulfilling (Rosso et al., 2010). Steger et al. (2012) describe calling as “a more specific construct that falls under the umbrella of meaningful work.” Calling is, therefore, a different construct from meaningful work and should not be used to measure meaningful work (Lips-Wiersma and Wright, 2012).

We also found instruments that measured meaningful work as a one-dimensional construct, such as the “meaning” subscale of the Psychological Empowerment Scale (PES) (Spreitzer, 1995) and the Spirituality at Work Scale (SWS) (Ashmos and Duchon, 2000). The “meaning” subscale of the PES consists of three items and reflects the degree to which people find their work to hold personal meaning, significance, or purpose. This subscale was originally taken from the research of Tymon (1988, Unpublished) on empowerment (Spreitzer, 1995). The “meaning at work” subscale of the SWS consists of seven items. Although the SWS considers meaningful work to be a one-dimensional construct, two dimensions can be identified in the scale: (1) the degree to which people find their work to hold personal meaning, significance, or purpose and (2) the contribution or benefit of work for others.

We included only scales that considered meaningful work as a multidimensional construct. The four scales included in this review are: (1) the Comprehensive Meaningful Work Scale (CMWS); (2) the Work And Meaning Inventory (WAMI); (3) the Meaningful Work Scale (MWS); and (4) the Meaning In Work Scale (MIWS). **Tables 2**, **3** provide an overview of the characteristics of the four included meaningful work scales.

**Table 2:** Operationalized dimensions of meaningful work in the scales.

Scales	Comprehensive Meaningful Work Scale (CMWS) Lips-Wiersma and Wright (2012)	The Work And Meaning Inventory (WAMI) Steger et al. (2012)	Meaningful Work Scale (MWS) Bendassolli et al. (2015)	Meaning in Work Scale (MIWS) Lee (2015b)
Definitions	MW is an individual subjective experience of the existential *significance* or *purpose* of work.	This paper adopts Rosso et al.’s (2010) definition of MW: work that is both *significant* and *positive* in valence (meaningfulness) and adds: the positive valence of MW has a eudemonic (growth- and purpose-oriented) rather than hedonic (pleasure-oriented) focus.	MW is *an effect of the coherence* between the characteristics one pursues and the characteristics he/she identifies at the work he/she does.	MW is the discovery of existential meaning from experiencing *positive emotion, finding meaning from work, and pursuing purpose or goals in the workplace.*
Subscale	Developing and becoming self	Meaning making	Moral correctness Expressiveness and identification at work.	Work as a part of life toward meaningful existence *Significance of work related to life Work toward meaningful existence Experienced an authentic self in work.*
Subscale	Expressing full potential		Autonomy Development and learning	
Subscale	Unity with others		Quality of working relationships	
Subscale	Service to others	Greater good	Work utility	Meaningful purpose and goals of work *Work purpose Work goals.*
Subscale	Inspiration			
Subscale	Reality			
Subscale	Balance			
Subscale				Meaning from work itself *Significance of work itself Work values Work orientation.*
Subscale		Positive meaning in work		Experienced positive emotion in work *Meaningfulness in work A sense of worth in work Self-fulfilling in work.* Meaningful purpose and goals of work *Work purpose Work goals.*
In alignment with proposed definition?	Yes, the result of fit perspective	Yes, the subjective experience perspective	Yes, the result of fit perspective	Yes, the subjective experience perspective


**Table 3:** Characteristic of the scales used in the review.

Title and author	Study characteristics	No. of scale items and scoring method	Psychometric characteristics
Comprehensive Meaningful Work Scale (CMWS) Lips-Wiersma and Wright (2012)	*N* = 275	28-item scale using a five-point Likert Scale.	*Construct validity*
	*Sector*		Convergent
	Various organizations		Copenhagen Psychosocial Questionnaire, subscale meaning of work *r* = 0.69, *p* < 0.001
	*Gender*		Existential Meaning of Work Scale, work as enabling self *r* = 0.17, *p* < 0.001
	Male: 44%		Existential Meaning of Work Scale, work as inhibiting selfhood *r* = -0.37, *p* < 0.001
	Female: 56%		Divergent
	*Age*		Meaning in Life Questionnaire *r* = 0.19, *p* < 0.001
	Mean age: 37.9		Neoclassical Calling Scale *r* = 0.56, *p* < 0.001
	*Education level* 75% post-high school education		Work Engagement Scale *r* = 0.71, *p* < 0.001
			Work Values Scale *r* = 0.34, *p* < 0.001
			Work Preference Inventory *r* = 0.34, *p* < 0.001
			CFI = 0.972
			RMSEA = 0.059
			*Internal reliability*
			α = 0.72 to 0.92
The Work And Meaning Inventory (WAMI) Steger et al. (2012)	*N* = 370	10-item scale using a five-point Likert Scale.	*Construct validity*
	*Sector*		Convergent
	Employees of one Western university		The Brief Calling Scale range subscales *r* = 0.42 to *r* = 54, *p* < 0.001
	*Gender*		Work orientation range subscales *r* = 0.49 to *r* = 61, *p* < 0.001 Divergent
	Male: 30.3%		No analyses
	Female: 69.7%		CFI = 0.96
	*Age*		RMSEA = 0.09
	Mean age: 44.6		*Internal reliability*
	*Education level*		α = 0.82 to 0.93
	Mean 9.4 years of education past 8th grade		
Meaningful Work Scale (MWS) Bendassolli et al. (2015)	*N* = 446	25-item scale using a six-point Likert Scale.	*Construct validity*
	*Sector*		Convergent
	Professionals working in creative industries in		No evidence/no analyses
	Brazil		Divergent
	*Gender*		No evidence/no analyses
	Male: 44.8%		CFI = 0.942
	Female: 55.2%		RMSEA = 0.057
	*Age*		*Internal reliability*
	Mean age: 29.7		α = 0.79 to 0.88
	*Education level*		
	Unknown		
Meaning In Work Scale (MIWS) Lee (2015b)	*N* = 158	25-item scale using a five-point Likert Scale.	*Construct validity*
	*Sector*		Convergent
	Nurses in acute-care hospital settings working		No evidence/no analyses
	full-time (36 h/week) in United States		Divergent
	*Gender*		No evidence/no analyses
	Male: 12%		CFI = 0.907
	Female: 86,7%		RMSEA = 0.08
	Missing 1.35%		*Internal reliability*
	*Age*		α = 0.91 to 0.95
	Mean age: 43.2		
	*Education level*		
	Diploma in nursing 4.4%		
	Associate degree 41.1%		
	Bachelor’s degree 42.4%		
	Master’s degree 3.8%		
	Doctorate degree 0.0%		
	Missing/other 8.2%		


#### General Scale Characteristics

The number of items in the meaningful work scales ranged from 10 to 28. Items were scored on a five- or six-point Likert Scale. In all studies in our final selection, the researchers identified dimensions of meaningful work based on empirical or literature studies and operationalized these dimensions into subscales in the meaningful work scales. As such, the *a priori* theoretical frameworks provided the structure of the instrument. The scales measured meaningful work as a three- or four-dimensional construct through corresponding subscales. The subscales of the meaningful work scale were partially overlapping. All scales measured the purpose or significance of work (service to others, greater good, meaning from work itself, significance of work itself) and the authenticity of the self (developing and becoming self, meaning making, moral correctness, expressiveness and identification at work, work as a part of life toward meaningful existence, significance of work related to life, work toward meaningful existence, experienced an authentic self in work, see **Table 2**).

#### Definition-Measurement Alignment

In the first study, we proposed an integrative and comprehensive definition of meaningful work. Evaluating the complete alignment of scales with this integrative definition, we found no match. We found that the WAMI and the MIWS are aligned with “the subjective experience of existential significance.” Particularly the Positive meaning subscale (e.g., I understand how my work contributes to my life’s meaning) of the WAMI and the subscale Experienced positive Emotion in Work of the MIWS (e.g., I have a good sense of what makes my job meaningful) are aligned with “The subjective experience of existential significance.” These subscales contained seven similar items, which is no coincidence, as the WAMI has been partially used to develop the MIWS (Lee, 2015b). The WAMI captures the experience of meaningful work, whereas the MIWS captures the experience as well as the existential significance of work in life (see **Table 2**).

We found that the CMWS and the MWS are aligned with “the features contributing to the fit between the individual and work.” The CMWS considers “developing and becoming self,” “expressing full potential,” “unity with others,” and “service to others” as constituents of meaningful work, while the MWS considers “moral correctness,” “expressiveness and identification at work,” “autonomy,” “development and learning,” “quality of working relationships,” and “work utility” as constituents of meaningful work. The CMWS and the MWS contain similar subscales, but the MWS subscales measure work characteristics (“my job is useful to society”; “my job allows me to develop”), whereas the CMWS subscales measure the fit between the individual and work features or experienced fulfillment of dimensions (“what we do is worthwhile”; “I am excited by the opportunities available to me”). In addition, the CMWS also measures the balance between these dimensions through three factors: reality, spirituality, and the balance between I/others and doing/being.

#### Reliability and Validity

The CMWS, WAMI, and MWS scales were validated in studies sized *N* = 275 or more. The MIWS (Lee, 2015b) was validated in a study sized *N* = 158. Hu and Bentler (1999) argued that a study sample of 250 or larger is necessary for validation purposes. The sample used to validate the MIWS, therefore was considered too small. All subscale reliabilities (Cronbach’s Alpha) were 0.72 or higher. The subscale reliabilities, therefore, were acceptable (Peterson, 1994).

We found that convergent validity was only examined for the CMWS and the WAMI; for the CMWS, correlations with the Copenhagen Psychosocial Questionnaire, subscale Meaning of Work (*r* = 0.69) and Existential Meaning of Work Scale were examined (subscales *r* = 0.17 to *r* = 0.37). For the WAMI, correlations with scores on the Brief Calling Scale (subscales *r* = 0.42 to *r* = 54) and Scale for Work Orientation (subscales *r* = 0.49 to *r* = 0.61) were examined. Carlson and Herdman (2012) suggested values of *r* = 0.70 or higher for acceptable convergent validity, the CMWS and the WAMI showed convergent validity values that fall short of this criterion. We found that divergent validity was only examined for the CMWS; its correlation with scores on the Meaning in Life Questionnaire (a non-work scale, *r* = 0.19), Neoclassical Calling Scale (*r* = 0.56), Work Engagement Scale (*r* = 0.71), Work Values Scales (*r* = 0.34), and Work Preference Inventory (*r* = 0.34) were examined. Values of *r* = 0.30 or lower can be considered as acceptable for divergent validity, and *r* = 0.30 or lower is viewed as a weak correlation (Hinkle et al., 2003). The CMWS only showed a correlation below *r* = 0.30 with the Meaning in Life questionnaire; correlations with the others scales where higher than *r* = 0.30.

All instruments we reviewed had CFI values of 0.90 or higher. The CMWS and the MWS had an RMSEA value of 0.59 or less. The WAMI had an RMSEA value of 0.09. The MIWS had a value of 0.08. Hu and Bentler (1999) consider CFI values of 0.90 or higher and RMSEA values of 0.06 or less acceptable for results of confirmatory factor analyses. Only two instruments, therefore, the CMWS and the MWS, met the criterion of having a CFI value of 0.90 or higher and an RMSEA value of 0.06 or less. The other instruments met the level of acceptance of CFI value but not RMSEA value. See **Table 4** for an overview of the methodological quality findings.

**Table 4:** Ratings for each of the scales included in the review (X if criteria met and 0 if not).

Title and author	Study sample 250 or more	Convergent *r* = 0.70 or higher	Discriminant *r* = 0.30 or lower	CFI value of 0.90 or higher	RMSEA value of 0.06 or less	Reliability scores above 0.7
Comprehensive Meaningful Work Scale (CMWS) Lips-Wiersma and Wright (2012)	X	0	X/0	X	X	X
Work And Meaning Inventory (WAMI) Steger et al. (2012)	X	0	0	X	0	X
Meaningful Work Scale (MWS) Bendassolli et al. (2015)	X	0	0	X	X	X
Meaning In Work Scale (MIWS) Lee (2015b)	0	0	0	X	0	X


### Discussion

At the beginning of this article, we argued that the conceptual ambiguity surrounding the construct of meaningful work has also made its mark on the scales that are available to measure the construct. We found that studies still use meaningful work scales that measure meaningful work as a one-dimensional concept or measure concepts that are similar to yet different from meaningful work (e.g., Scroggins, 2008; Bunderson and Thompson, 2009; Pradhan and Pradhan, 2016).

The recent use of one-dimensional scales can perhaps be explained by the fact that empirical studies often do not use definitions of meaningful work that are based on an underling theoretical framework. Without consensus in the field on how to measure meaningful work, chances are that available scales are used as an ad hoc measure without being driven by a comprehensive definition or underling theoretical framework. Rosso et al. (2010) observe, furthermore, that meaningful work is frequently approached as a one-dimensional construct, although recent research suggests it is a multidimensional construct. As a result, available scales of meaningful work have non-specific items such as “the work that I do is meaningful to me” (Spreitzer, 1995); “I experience joy in my work” (Ashmos and Duchon, 2000); “the work I do on this job is very important to me” (May et al., 2004); “Life is most worth living when I am absorbed in work” (Fairlie and Flett, 2004); and “my job is very significant and important in the broader scheme of things” (Rafferty and Restubog, 2011). Another scale frequently used to measure meaningful work is the meaningfulness subscale of the Job Diagnostic Survey or JDS (Hackman and Oldham, 1975). The JDS uses two pairs of items referring to the respondents’ personal feelings and their perceptions of their co-workers’ feelings about whether job tasks seem useless and whether their work is meaningful.

Although most of these scales have acceptable reliabilities, the non-specific items raise the question what they actually measure (Steger et al., 2012). The items “the work that I do is meaningful to me” of Spreitzer (1995); “I experience joy in my work” (Ashmos and Duchon, 2000); and “the work I do on this job is very important to me” (May et al., 2004), for example, can be interpreted as work being important rather than work being a reason for being. The other two items of Fairlie and Flett (2004) and Rafferty and Restubog (2011) are more related to the role of work in one’s life rather than what is meaningful in work. The simplicity of the scales limits their explanatory potential. These scales, therefore, are not precise enough to adequately distinguish antecedents to and outcomes from multiple dimensions contributing to meaningful work or to understand their complex interplay (Lips-Wiersma and Wright, 2012; Steger et al., 2012).

None of the reviewed scales are completely aligned with the full-proposed definition. However, we found that the WAMI align with experience of meaningful work, and the CMWS align with features of work and individual contributing to the fit between the individual and work. These findings suggest that a scale can be developed that fully aligns with our proposed definition of meaningful work by integrating the above- mentioned scales.

By assessing the methodological qualities of the scales, we found that none of the scales had good evidence of convergent and divergent validity. However, we found that the WAMI and the CMWS showed values of acceptable model fit. Based on the evaluation of alignment and quality assessment, we argue for using the WAMI for studies aiming to examine the relations between the experience of meaningful work and certain antecedents or outcomes. The WAMI was specifically developed to capture the multidimensional experience of meaningful work. We observed that a number of experimental studies from recent years have used the WAMI (e.g., Allan et al., 2016; Tavares, 2016; Tims et al., 2016), which is encouraging for the field.

We argue for using the CMWS for studies aiming to improve our understanding of the way in which personal characteristics, task activities, and organizational practices create meaningful work. The CMWS has three additional subscales (reality, spirituality, and balance) to capture the dynamic interplay between the dimensions. As such, the CMWS is more suitable than the MWS – which views work as a static rather than a dynamic process – to explain the complex interplay between the dimensions and relations to antecedents and outcomes.

In conclusion, we evaluated existing meaningful work scales in light of our proposed definition and assessed their psychometrics characteristics. We argue for using two scales. In the next section, we will discuss the results, limitations, and implications of both studies together.

## General Conclusion and Discussion

Although previous reviews of meaningful work have increased coherence in the disparate literature on meaningful work, this article reveals that the construct of meaningful work is still defined and, hence, measured in suboptimal ways. For the meaningful work research field to mature scientifically, conceptualization and measurement efforts should begin to coalesce rather than diverge. The objectives of this article were to establish an integrative and comprehensive definition of meaningful work and to evaluate existing meaningful work scales in light of this definition. Therefore, we conducted two literature studies on definitions and scales of meaningful work.

Based on the results of the first literature study we propose the following integrative and comprehensive definition: meaningful work is the subjective experience of existential significance resulting from the fit between the individual and work. The “subjective experience of existential significance” refers to the process of personally perceiving work as contributing to, or making sense of, one’s reason for existence in the world. The “result of the fit” refers to the fulfillment of dimensions – inherent in every human being – through or in work. These dimensions should be defined further by the underlying conceptual framework. Based on the results of the second literature study we have identified two validated scales that align with this definition and have been validated: the WAMI and the CMWS. Using these scales could create greater consistency and integration of results in the field. Therefore, we argue that these two scales should be considered as appropriate scales for the future research in the field regarding paid work. We suggest that the WAMI and the CMWS might be integrated into one scale in order to have a single scale that is fully aligned with the proposed definition.

### Limitations of Both Studies

There are two limitations to this article we would like to discuss. The first limitation concerns the subjective categorization of the definitions and the subjective evaluation of alignment with the proposed definition. The categories have been identified and alignment has been evaluated by the first author herself, potentially reflecting a subjective view. However, the categorization and alignment were discussed among the three authors to alleviate this concern.

The second limitation concerns the exclusion of scales that were not validated. Because the focus of this systematic review was on meaningful work scales for which psychometric properties had been reported, studies that used a meaningful work scale but did not report instrument validation or psychometric analysis were not included (e.g., Fairlie and Flett, 2004). Studies that reported validation of meaningful work scales in languages other than English were not included either (e.g., Morin, 2003; Höge and Schnell, 2012). Methodological quality assessment of these scales would be a valuable step in introducing greater coherence into the field of meaningful work.

### Avenues for Further Research

Furthermore, in this article we viewed work in the context of paid work. An interesting avenue for future research is to explore the applicability of our findings within a more holistic context, encompassing for example family-related tasks or volunteering.

In this article, we presented a systematic review of the instruments used to measure meaningful work. We did not conduct new validation studies. Although our findings provide some insights into the convergent and divergent validity of meaningful work scales, additional work is needed to understand the validity of these scales more fully.

Finally, we urgently call for using a comprehensive definition of meaningful work and corresponding validated meaningful work scales in empirical studies in paid work contexts, so results can be compared and consensus can be reached. We argue that achieving consensus on using existing scales instead of developing new ones will greatly facilitate the development of the field of meaningful work.

## Author Contributions

JB-N designed the literature studies, carried out the literature studies, analyzed the data, and drafted the manuscript. MD and BB have made substantial contributions to interpretation of data and have helped to draft the manuscript and revise it critically for important intellectual content. All authors have read and approved the final manuscript. All authors agreed to be accountable for all aspects of the work.

## Conflict of Interest Statement

The authors declare that the research was conducted in the absence of any commercial or financial relationships that could be construed as a potential conflict of interest.
